# BATMAN-TCM: a Bioinformatics Analysis Tool for Molecular mechANism of Traditional Chinese Medicine

**DOI:** 10.1038/srep21146

**Published:** 2016-02-16

**Authors:** Zhongyang Liu, Feifei Guo, Yong Wang, Chun Li, Xinlei Zhang, Honglei Li, Lihong Diao, Jiangyong Gu, Wei Wang, Dong Li, Fuchu He

**Affiliations:** 1 State Key Laboratory of Proteomics, Beijing Proteome Research Center, Beijing Institute of Radiation Medicine, Beijing 100850, China; 2 National Center for Protein Sciences Beijing, Beijing 102206, China; 3 Institute of Basic Medical Sciences Chinese Academy of Medical Sciences, School of Basic Medicine Peking Union Medical College, Beijing 100005, China; 4 Beijing University of Chinese Medicine, Beijing 100029, China; 5 Beijing Genestone Technology Ltd., Beijing 100085, China; 6 Beijing National Laboratory for Molecular Sciences, State Key Lab of Rare Earth Material Chemistry and Applications, College of Chemistry and Molecular Engineering, Peking University, Beijing 100871, China

## Abstract

Traditional Chinese Medicine (TCM), with a history of thousands of years of clinical practice, is gaining more and more attention and application worldwide. And TCM-based new drug development, especially for the treatment of complex diseases is promising. However, owing to the TCM’s diverse ingredients and their complex interaction with human body, it is still quite difficult to uncover its molecular mechanism, which greatly hinders the TCM modernization and internationalization. Here we developed the first online Bioinformatics Analysis Tool for Molecular mechANism of TCM (BATMAN-TCM). Its main functions include 1) TCM ingredients’ target prediction; 2) functional analyses of targets including biological pathway, Gene Ontology functional term and disease enrichment analyses; 3) the visualization of ingredient-target-pathway/disease association network and KEGG biological pathway with highlighted targets; 4) comparison analysis of multiple TCMs. Finally, we applied BATMAN-TCM to Qishen Yiqi dripping Pill (QSYQ) and combined with subsequent experimental validation to reveal the functions of renin-angiotensin system responsible for QSYQ’s cardioprotective effects for the first time. BATMAN-TCM will contribute to the understanding of the “multi-component, multi-target and multi-pathway” combinational therapeutic mechanism of TCM, and provide valuable clues for subsequent experimental validation, accelerating the elucidation of TCM’s molecular mechanism. BATMAN-TCM is available at http://bionet.ncpsb.org/batman-tcm.

Traditional Chinese Medicine (TCM), with a history of thousands of years of clinical practice, plays an important role in maintaining the health of peoples in Asia, and is gaining more and more application and attention all over the world^1^,^2^,^3^,^4^. For example, the journal of *Science* has published a special supplement with a series of reports and articles for TCM in Dec. 2014^4^. TCM research is also promising for the future new drug development, especially for the treatment of complex diseases such as cancers, diabetes etc. because of its advantage of “multi-component, multi-target and multi-pathway” combinational regulatory mechanism^5^. For example “Fufangdanshen dripping Pill” for chronic stable angina (ClinicalTrials.gov ID of FDA: NCT00797953) and “Fuzheng Huayu” for chronic hepatitis C patients with hepatic fibrosis (ClinicalTrials.gov ID of FDA: NCT00854087) have completed Phase II clinical assessments. And TCMs have become important natural template library for new drug development. For example, several famous drugs derived from TCMs include Ephedrine (DrugBank ID: DB01364) extracted from *Ephedra Sinica Stapf* for asthma treatment, Arsenic trioxide (DB01169) for acute promyelocytic leukemia, and Artemether (DB06697) from *Artemisia Carvifolia* for malaria whose discoverer won the Nobel Prize in Physiology or Medicine in 2015 and so on. And now in China, more than 70 drugs developed based on TCMs have been widely applied in clinic (China FDA website: http://www.sfda.gov.cn/WS01/CL0001/).

However, TCM is different from the modern western medicine in substance, methodology and philosophy^1^, hindering western countries from recognizing the TCM. Therefore, to make TCM serve people all over the world better and accelerate the promising TCM-based new drug development, it’s necessary to bring the ancient practice of TCM into line with modern standards^2^,^6^, among which the elucidation of the molecular mechanism of TCM is one of the most important issues. However, owing to the diversity of TCM’s ingredients and the complexity of the interaction between TCM and the human body, it is still quite difficult to uncover the mechanism underlying TCM. The clarification of the TCM’s molecular mechanism has become a bottleneck in TCM modernization and internationalization, which is urgent to be solved.

Recently, as the emergence of databases for herbal ingredients (e.g. TCM-ID^7^, TCM Database@Taiwan^8^, TCMID^6^) and the development of compound-target interaction prediction methods [e.g. 9,10,11,12], more and more evidence indicates that combining target prediction of herbal ingredients and subsequent network pharmacology analyses is a feasible and powerful way to analyze the molecular mechanism of TCM^13^,^14^,^15^,^16^,^17^,^18^. For example, Li *et al.* combined a pharmacophore modeling technique and docking to identify potential targets of Danshen Formula’s ingredients and further studied Danshen Formula’s mechanism for cardiovascular disease treatment by constructing and analyzing compound-target-pathway-disease networks^14^. Liu *et al.* and Li *et al.* both used the method that integrates the chemical, genomic and pharmacological information^19^ to predict targets of potential bioactive components of TCM and then analyzed compound-target-disease networks to respectively uncover Licorice’s and Eucommia ulmoides Oliv.’s mechanisms^15^,^16^. Specially, in our previous jobs, we combined drug structure-based target prediction and network/biological pathway-based analyses of potential targets together with following experimental validation to successfully elucidate the molecular mechanism of Qishenkeli acting on the coronary heart disease^17^ and DanQi Pill against heart failure^18^.

Now there have been several databases which store herbal ingredients’ target information including HIT^20^, TCMID^6^ and recently developed TCMSP^21^, and TCMID and TCMSP even provide herbal ingredient-target-disease network visualization, which indeed contribute to the mechanism research of TCM. However, for TCM’s molecular mechanism study, these databases are far from enough. The reasons include: 1) These databases are not comprehensive enough. They only store limited number of TCM formulas or herbs from manual curation, and many formulas or herbs are not covered. 2) They cannot support user-customized input of a formula/herb’s ingredient list. This function of customized input is usually very useful, facilitating the flexible input of active ingredients, metabolites of original constituents, ingredients detected in plasma or something like those that really contribute to its therapeutic effects based on other prior knowledge. 3) They don’t support the integration analyses of all ingredients of a formula/herb, which is crucial for understanding TCM’s holistic mechanism comprehensively: on one hand, for a majority of ingredients without known targets they lack online target prediction, and TCM’s holistic mechanism cannot be well explored only by analyzing known targets of partial ingredients (Unlike western small-molecule drugs, most herbal ingredients’ targets are unknown. For example, even by comprehensive literature curation, only 6 percent of 9862 herbal ingredients in HIT have been found to have direct or indirect targets in PubMed abstracts^20^); on the other hand, they lack the function of integration analyses of all ingredients’ targets. Therefore, an online analysis tool which supports user-customized input and interactive analysis functions is necessary for TCM’s molecular mechanism research.

Here we developed the first online bioinformatics analysis tool specially designed for the research of molecular mechanism of TCM - BATMAN-TCM (a Bioinformatics Analysis Tool for Molecular mechANism of Traditional Chinese Medicine), which was based on TCM ingredients’ target prediction and subsequent network pharmacology analyses. This tool will contribute to the understanding of the therapeutic mechanism of TCM (“multi-component, multi-target and multi-pathway”), and provide clues for the following experimental validation.

## Results

### Performance evaluation of the target prediction method

Unlike western drugs, most herbal compositive compounds’ targets are unknown. Therefore to understand the TCM’s molecular mechanism comprehensively and systematically, the function of target prediction in BATMAN-TCM is necessary.

Here a similarity-based target prediction method was adopted. The core idea of this method, which was first proposed by Perlman *et al.*^22^, is to rank potential drug-target interactions based on their similarity to the known drug-target interactions (see Materials and Methods).

**Figure 1:**
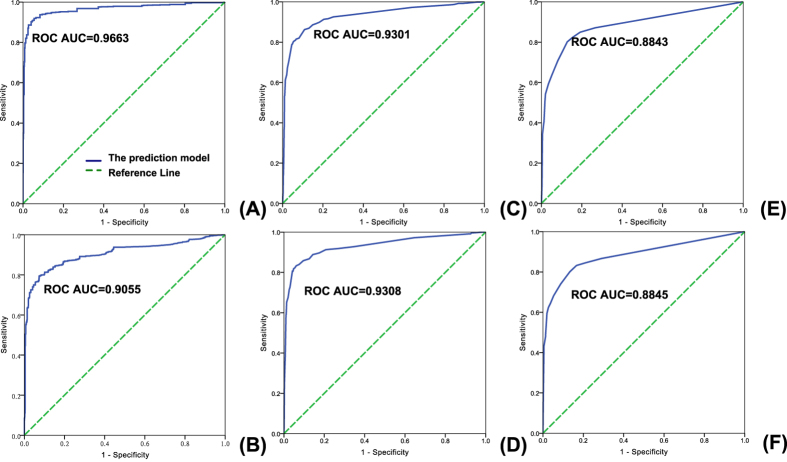
ROC curves and their corresponding AUCs of the target prediction model based on cross-validation and the independent test set. (**A**,**B**) ROC curves and their AUCs based on the golden standard dataset using leave-one-interaction-out cross-validation (**A**) and leave-one-drug-out cross-validation (**B**). (**C**) ROC curve and its AUC based on the independent test set which is independent of the golden standard dataset on drug-target interaction. (**D**) ROC curve and its AUC based on “the independent test set-drug” which is independent of the golden standard dataset on drug (see Materials and Methods). (**E**,**F**) ROC curves and their AUCs of the prediction model only integrating chemical structure-involved features based on the independent test set (**E**) and “the independent test set-drug” (**F**).

To evaluate the performance of the prediction model, first “leave-one-interaction-out” cross-validation scheme was used, in which each time a drug-target interaction was picked from the golden standard dataset to be used as the test interaction (see Materials and Methods). We found that our prediction model obtained excellent prediction performance (ROC AUC = 0.9663) (Fig. 1A). Unlike western drugs, most herbal ingredients have no known target information, and therefore in most cases, the target prediction of herbal ingredients is the “*ab initio*” prediction problem. To simulate this condition, “leave-one-drug-out” cross-validation was also used, where each time a drug together with its all (positive and negative) targets was chosen from the golden standard dataset to be used as the test set (see Methods and Materials). And our prediction model also showed good prediction ability based on the “leave-one-drug-out” cross-validation (Fig. 1B). Besides the cross-validation, we also used the independent test set to test the performance. Likewise, we used two independent test sets: one was independent of the golden standard dataset on drug-target interactions and the other was independent on drugs which was denoted as “the independent test set-drug” to simulate the “*ab initio*” prediction (see Materials and Methods). The results based on the two independent test sets further indicated that our method can be efficiently used to discriminate true compound-target interactions from others, and was powerful even for the “*ab initio*” prediction of targets (Fig. 1C,D). In addition, considering that for the vast majority of TCM compositive compounds chemical structures are the only known information, we used independent test set to specially assess the performance of the prediction model only integrating chemical structure-involved features including FP2-closeness, functional_group-sequence and functional group-GO (see Materials and Methods). In result, our model still had good prediction ability (Fig. 1E,F).

### Inputs of BATMAN-TCM

BATMAN-TCM supports three alternative input types and a special input type designed for comparison analysis. 1) TCM formula represented by Pinyin name (e.g. huo xiang zheng qi san); 2) Herb or herb list denoted by Pinyin name, English name or Latin name (e.g. ren shen, Ginseng or *Panax ginseng*), which is designed typically for the research of a herb or a formula composed of multiple herbs; or 3) Compound list denoted by PubChem_CID or chemical structure of InChI format, which is typically used for the study of a formula or a herb composed of multiple ingredients. The second and third input types enable user-customized analyses. By these two input types, users may analyze a formula or herb not deposited in our database backstage as long as the compositive herbs or ingredient list of the formula/herb is provided. Especially the third input type supports customized ingredient list, which may be TCM’s active ingredients, metabolites of original ingredients, ingredients detected in plasma or something like those that really contribute to its therapeutic effects supported by other prior knowledge. 4) Except the three input types above, BATMAN-TCM also allows users to simultaneously input multiple TCM formulas/herbs/compound lists for comparison/combinational analyses (see more details in the following part).

### Outputs of BATMAN-TCM

For user-submitted formula/herb, BATMAN-TCM first uniformly transfers it into a list of ingredients based on the database backstage, and then predicts potential targets of these ingredients and performs further bioinformatics analyses on these potential targets. BATMAN-TCM includes four main outputs as shown below.

#### 1) TCM ingredients’ predicted targets

**Figure 2:**
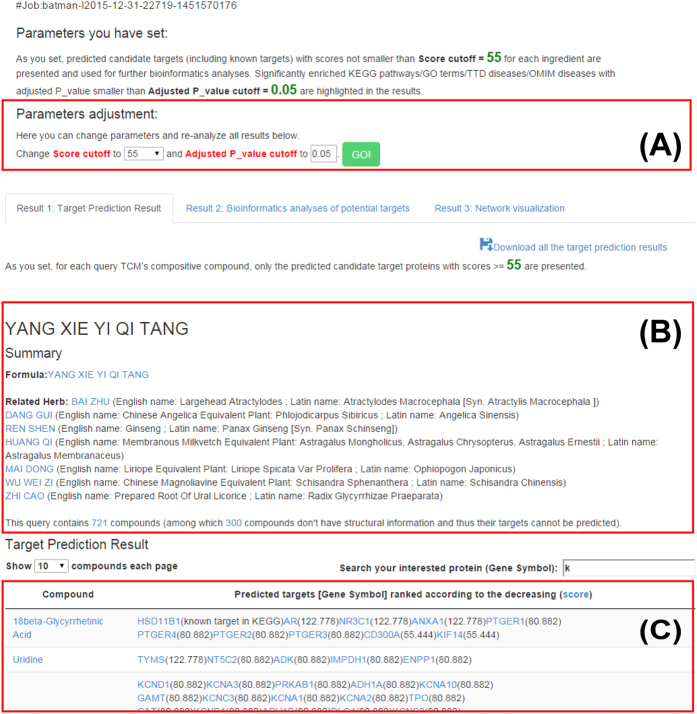
Target prediction result page. (**A**) “*Score_cutoff*” and “Adjusted P-value cutoff” (the cutoff of P-value after Benjamini-Hochberg multiple testing correction for enrichment analyses) can be changed on the result page. Once “*Score_cutoff*” or “Adjusted P-value cutoff” is changed, all analysis results will be updated. (**B**) A summary about the query, including formula, compositive herbs and ingredient list with hyperlinks to TCMID. (**C**) The target prediction result table. In this table, for each ingredient, known targets marked by “known target in DrugBank, KEGG or TTD” are given first if there are, followed by predicted targets ranked according to the decreasing scores given by our target prediction model. Ingredients are crosslinked to PubChem or TCMID and targets to GeneBank. Users can search their interested protein among the targets by the search box on the upper right corner.

TCM achieves its treatment effects by acting on multiple proteins, biological pathways in human body, and thus exploring TCM ingredients’ targets, target pathways/biological processes is crucial for uncover its molecular mechanism. For the query TCM’s each ingredient, the candidate target proteins whose scores given by our target prediction method exceed a given cutoff “*Score_cutoff*” (see Materials and Methods) and known targets are listed in the result table. These listed targets are considered as the “potential targets” and all the following analyses are based on these potential targets. The “*Score_cutoff*” can be set by users and also be adjusted on the result page (Fig. 2).

#### 2) Functional analyses of targets

**Figure 3:**
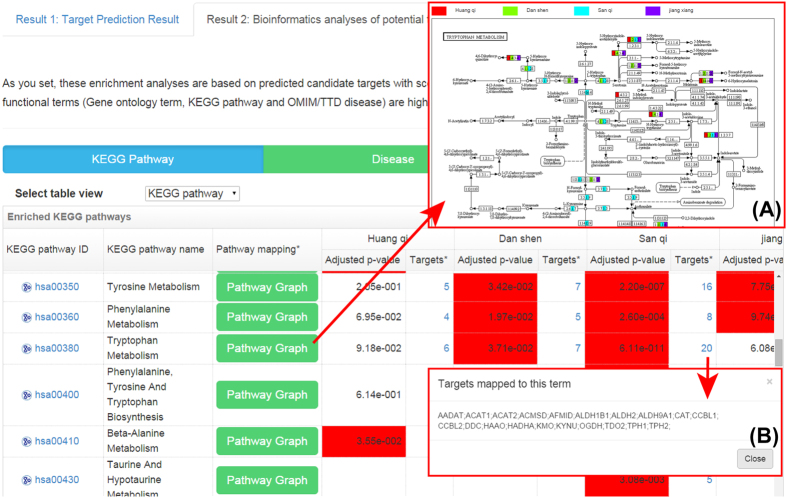
Target analysis result page. Significantly enriched KEGG pathways, GO functional terms and diseases together with adjusted P-value and targets mapped to this term are presented. The significantly enriched terms with adjusted P-value smaller than the cutoff set by users are highlighted in red. (**A**) Clicking on the “Pathway Graph” button in the KEGG pathway result table will lead to the pathway view with highlighted targets mapped on it (denoted by different colors for different clusters). (**B**) “Targets” are referred to as the targets mapped to the term, and clicking on the number will present the detailed target list.

The significantly enriched KEGG biological pathways, Gene Ontology (GO) functional terms (including biological process, molecular function and cellular component) and OMIM/TTD disease phenotypes among the query TCM’s potential targets together with corresponding adjusted P-value (i.e. P-value after Benjamini-Hochberg multiple testing correction) and targets mapped to this term are presented in the tables (Fig. 3). These significantly enriched biological pathways and GO functional terms may play crucial roles for TCM’s therapeutic effects, providing direct clues for further experimental validation of molecular mechanism of TCM. TCM is prescribed for particular “pattern” (“zheng” in Chinese), while modern western drugs are designed for treating particular disease^6^. The overrepresented diseases among the potential targets of TCM construct the connection between TCM and diseases by targets and disease-related genes, not only bridging the gap between TCM and modern western medicine but also providing clues for exploring novel diseases on which the TCM has therapeutic effects^6^.

Especially, clicking on the “Pathway Graph” button in the result table of the KEGG pathway enrichment analysis will lead to the pathway view with highlighted targets (Fig. 3A). The biological pathway view of the targets and their upstream/downstream relationship can help the deep analyses of TCM’s potential molecular mechanism and facilitate the design of further experimental validation.

#### 3) The visualization of ingredient-target-pathway/disease association network

**Figure 4:**
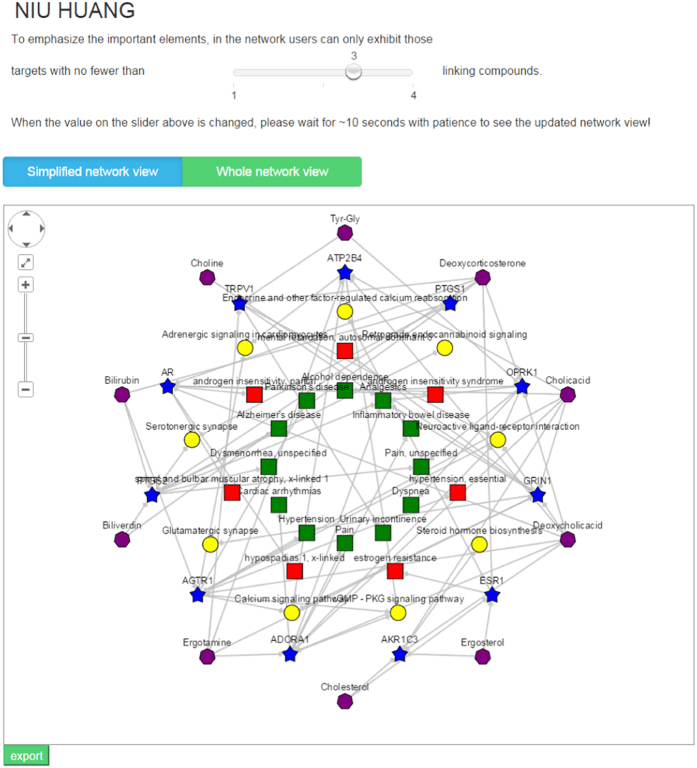
Ingredient-target-pathway/disease association network. In the association network, there are four kinds of nodes distinguished by different shapes and colors including TCM’s ingredients, targets, biological pathways and OMIM/TTD diseases and three types of edges including ingredient-target association, target-pathway association and target-disease association. In addition, to focus on the important elements, the size of the target node, pathway node and disease node is proportional to their degree in the network, and users can only show those targets with more than M (which can be adjusted by the slider) linking compounds in the network. Different from the “Whole network view”, in the “Simplified network view” only those significantly enriched pathways/diseases are shown in the network. The network graph can be exported as .PNG format.

In the view of ingredient-target-pathway/disease association network, the association relationships between the interested TCM’s ingredients and their known and potential targets, these targets and their located biological pathways, and these targets (/genes) and their related diseases are presented. Different types of nodes are presented by different node shapes and colors. In addition, to help users focus on the important network elements, the size of the target node, pathway node and disease node is proportional to their degree in the network, which is respectively defined as the number of compounds acting on the target, the number of targets involved in the pathway and the number of targets being known related genes of the disease, and meanwhile users can only exhibit those targets with more than “M” linking compounds (the value of M can be adjusted by the slider on the result page) in the network. Different from the “Whole network view”, in the “Simplified network view” only those significantly enriched pathways/diseases (adjusted P-value <  = cutoff set by users) are shown in the network (Fig. 4). The “ingredient-target-pathway/disease” association network helps understand the “multi-component, multi-target and multi-pathway” combinational therapeutic mechanism and the potential disease treatment mechanism of TCM in an intuitive way.

#### 4) Comparison analyses

Besides supporting the research of the molecular mechanism of a single formula or herb, BATMAN-TCM also provides the function of comparison/combinational analyses of multiple formulas/herbs by “Add one cluster” button on the homepage of BATMAN-TCM to allow users to submit multiple queries (i.e. multiple formulas/herbs/herb lists/compound lists). In this case, each query is defined as a “cluster” in BATMAN-TCM, and you can name the cluster by yourself to discriminate different clusters.

Formula, the main therapeutic concept in TCM, is a combination of multiple herbs which are organized based on the combinatorial principle of “emperor-minister-adjuvant-courier” (“jun-chen-zuo-shi” in Chinese)^3^,^13^,^23^. The “jun” herb treats the main cause or primary symptoms of a disease, the “chen” herb assists the “jun” herb to enhance its therapeutic effects and relieve accompanying symptoms, the “zuo” herb is often used to counteract the possible toxic or side-effects of other herbs and the “shi” herb generally ensures the absorption of the formula’s components and helps deliver or guide them to the target organs^3^,^23^. The comparison/combination analysis function is originally designed to be used for comparing different compositive herbs of a formula and helping understand this combinational principle of a formula from molecular and systematic level. And this function of BATMAN-TCM can also be used to compare any different formulas, herbs or compound lists for users’ own purposes.

When users input multiple queries, the corresponding combinational analysis results of the three outputs mentioned above will be given. For example for the target prediction, besides the target prediction result table for each query will be presented, a Venn graph will also be produced for the comparison between the target sets of different queries/clusters, in which the number of targets common to multiple clusters or specific to a cluster will be given and clicking on the different areas of the Venn graph will lead to the corresponding target list. For the “Pathway Graph” function on the pathway enrichment analysis result page, targets belonging to different clusters are mapped to the common KEGG biological pathway, which are highlighted by different colored nodes (Fig. 3A).

### Use case

In order to demonstrate the usage of BATMAN-TCM, we used the TCM formula - Qishen Yiqi dripping Pill (QSYQ) - as an example. QSYQ (Tasly Pharmaceutical Co. Ltd., Tianjing, China; Approval number of China FDA: Z20030139) is composed of four herbs including *Radix Astragali* (Huangqi in Chinese), *Radix Salvia miltiorrhiza* (Danshen), *Radix Notoginseng* (Sanqi) and *Lignum Dalbergia Odorifera* (Jiangxiang), which respectively act as “emperor”, “minister”, “adjuvant” and “courier” in the formula. In China QSYQ has been widely used for the clinical treatment of chronic cardiac insufficiency, angina pectoris, coronary heart disease etc.^24^,^25^,^26^, but its molecular mechanism is still not very clear.

To analyze its potential molecular mechanism, 109 compositive compounds of QSYQ from the book - *Modern Chinese Materia Medica*^27^ and HIT database^20^ were submitted to BATMAN-TCM (Supplementary Table S1 online). First the known and predicted targets were obtained by the retrieval in the integrated known drug-target interaction dataset (see Materials and Methods) and the prediction respectively. For the 109 ingredients we obtained 1463 potential targets (including 18 known targets) (Supplementary Table S2 online). Then enrichment analyses were implemented for them (Supplementary Table S3~S6 online). We found that biological pathway enrichment analyses revealed many QSYQ’s potential target pathways, many of which were known to play important roles in cardiac physiology and pathology. For example, 1) The neuroactive ligand-receptor interaction pathway (adjusted P-value = 5.23e-36) plays important roles in the development and progress of cardiovascular diseases, and in this pathway Adrenergic receptor, Angiotensin receptor etc. are closely related with cardiac function^17^,^28^,^29^,^30^; 2) Calcium signaling pathway (adjusted P_value = 2.09e-19) has also been reported to be related with heart failure and Calcium antagonists have been widely used to reduce intracellular Calcium concentration and further lower myocardial contractility^31^; 3) Aminoacyl-tRNA biosynthesis (adjusted P-value = 3.06e-12) is associated with cardiac angiogenesis^32^; 4) Activated renin-angiotensin system (RAAS) (adjusted P-value = 4.70e-02) is closely related with cardiovascular function deterioration^33^. Significantly enriched disease phenotypes included analgesics (adjusted P-value = 2.41e-12), hypertension (1.50e-07), heart failure (4.13e-04), congestive heart failure (6.95e-04), cardiovascular disease_unspecified (1.42e-03), cardiac arrhythmias (3.45e-03) and vascular disease (4.28e-02) etc., many of which are QSYQ’s known indications and other suggest QSYQ’s potential therapeutic effects (all the analysis results can be interactively browsed by the URL: “ http://bionet.ncpsb.org/batman-tcm/index.php/home/jobquery/index/jobId/batman-I2015-12-31-68977-1451525385”).

**Figure 5:**
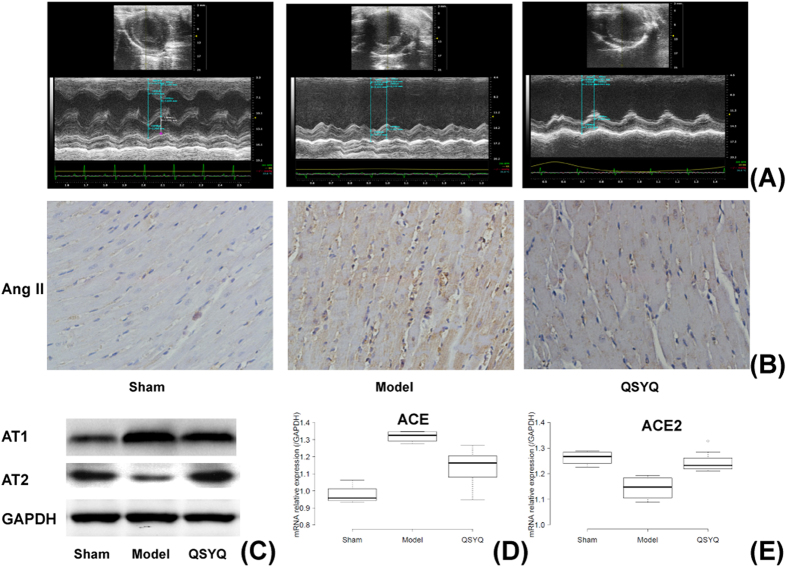
Experimental validation of the RAAS pathway targeted by QSYQ based on HF model of SD rats. (**A**) Echocardiography results reflecting apparent different cardiac functions (described by EF) and ventricular wall movement (described by LVEDs and LVEDd) of rats in different groups. (**B**) IHC results of Ang II in myocardial tissues of rats in different groups. (**C**) The expression levels of AT1 and AT2 in different groups based on western blot. (**D**,**E**) The expression levels of ACE and ACE2 in different groups based on PCR. The boxplots were draw by BoxPlotR^56^.

As mentioned above, QSYQ has been widely used for the clinical treatment of cardiac diseases and has a definite cardioprotective effect, and the relationship between RAAS pathway and the cardiovascular function is also well known. However the relationship between the cardioprotective effect of QSYQ and RAAS has not been reported yet. Here for the predicted RAAS pathway, we performed further experimental validation. Previous study has indicated that in this pathway the up-regulation of angiotensin II (Ang II) together with its receptor AT1 significantly contributes to the deterioration of cardiovascular function, and antagonists to AT1 and inhibitors of angiotensin-converting enzyme (ACE) which hydrolyzes Ang I to Ang II have been routinely used to treat patients with coronary heart disease^34^. Recently the cardioprotective effects of ACE2 and AT2 in this pathway were also identified. ACE2 metabolizes Ang II into Ang 1-7, acting as an endogenous antagonist of Ang II^34^, and the overexpression of AT2 was found to partially reverse the myocardial infarction-induced cardiac dysfunction^35^. To study the effect of QSYQ on RAAS, we respectively built the HF (heart failure) model group, QSYQ group and the sham-operated group using SD (Sprague-Dwaley) rats (see Materials and Methods). First we compared the cardiac function of rats from the three groups based on echocardiography’s ejection fraction (EF) parameter, which is referred to as the fraction of blood pumped from the heart each heartbeat and serves as a general measure of the cardiac function. In result we saw that compared with that of the sham-operated group, the EF of rats from the HF model group was substantially reduced to <50%, indicating HF model was successfully constructed according to the clinical diagnosis criterion of HF. And after QSYQ treatment (i.e. QSYQ group v.s. HF model group) the EF obviously recovered, indicating QSYQ can efficiently restore the injured cardiac function (Fig. 5A). Further we found that compared with the sham group, in model group ACE, Ang II and AT1 were greatly up-regulated (t-test, P-value = 5.2e-05 for ACE, 0.021 for Ang II and 0.004 for AT1), while the expression of AT2 and ACE2 with cardioprotective effects significantly decreased (P-value = 0.007 for AT2 and 0.002 for ACE2). QSYQ can down-regulate ACE (QSYQ group v.s. model group, P-value = 0.006) and up-regulate ACE2 (P-value = 0.002) to reduce the expression of Ang II (P-value = 0.034), and further combine with the inhibition of the expression of AT1 (P-value = 0.005) to blocks the activation of RAAS. Meanwhile QSYQ can also up-regulate AT2 (P-value = 0.005) to achieve the cardioprotective effects (Fig. 5B–E).

Besides the holistic analyses of the formula, we also performed the comparison analyses of QSYQ’s four compositive herbs (interactively browse the analysis results by: “ http://bionet.ncpsb.org/batman-tcm/index.php/home/jobquery/index/jobId/batman-I2015-12-31-92282-1451525557”), and obtained some interesting results. For example, from disease enrichment analyses, we see that the effects of emperor drug Huangqi are mainly related with cardiac disease phenotypes including heart failure, ischemic heart disease, pain, while besides some phenotypes which overlap with emperor drug Huangqi’s, the significantly enriched diseases of minister drug Danshen also include vascular indications such as hypertension, vascular disease which are the common accompanying pathological changes of cardiac disorders. The enriched diseases of assistant drug Sanqi and courier drug Jiangxiang highly overlap with those of emperor drug and minister drug. These results suggest Huangqi as the emperor mainly treats the heart dysfunctions, while Danshen as the minister protects the vessel synergeticly and meanwhile assist the emperor together with assistant and courier drugs to achieve the cardiovascular protection effects.

In summary, here we took QSYQ as an example to show the functions of BATMAN-TCM, and further combined with subsequent experimental validation to elucidate that QSYQ as a whole regulates different subtypes of ATs and ACEs in RAAS to achieve its cardioprotective effects in HF rat models for the first time, indicating BATMAN-TCM is a powerful tool for TCM molecular mechanism research.

## Discussion

Here we developed the first bioinformatics analysis tool for molecular mechanism of TCM - BATMAN-TCM. Compared with current TCM databases such as HIT, TCMID and TCMSP, besides integrating known formula/herb-ingredient-target-disease association information, as an analysis tool BATMAN-TCM provides multiple unique analysis functions, including 1) Online target prediction for ingredients without targets in database backstage; 2) Integration analyses of each ingredient’s (potential) targets; 3) Comparison analyses of multiple TCMs. BATMAN-TCM also supports the input of customized ingredient list and enables the analysis of a formula/herb not in the database backstage. These analysis functions will generate new knowledge absent in both current databases and literature mining, and significantly contribute to the comprehensive understanding of a formula/herb’s holistic mechanism.

Besides the functions stated above, there is one more function in BATMAN-TCM which we call “Function2TCM”. By this function, users can retrieve the TCM formulas and herbs significantly associated with a certain GO/KEGG_pathway/OMIM or TTD disease term. This function is implemented based on BATMAN-TCM’s analysis results for TCMID formulas/herbs (see Materials and Methods). Clicking on the “Function2TCM” in the navigation bar at the top of BATMAN-TCM homepage will lead to the Function2TCM page.

To show the usage and usefulness of BATMAN-TCM, QSYQ-RAAS was used as an example. The experimental validation of QSYQ’s effect on RAAS has original contribution to the elucidation of QSYQ’s mechanism. The relationship between RAAS and the deterioration of cardiovascular function is well known^33^, however, the relationship between QSYQ formula as a whole and RAAS has not been previously reported and validated. As far as we know, currently only *Panax notoginseng saponin*, an active fraction of *Radix notoginseng*, has been reported to up-regulate ACE2 expression in acute myocardial infraction rats *in vivo*^36^ and inhibit human endothelium cells’ damages induced by AngII via AT1 receptor *in vitro*^37^. However, it is well known in the TCM research field that when they cooperate with other fractions in the formula as a whole, the mechanism of active fractions or monomers is not necessary to be the mechanism of TCM formula as a whole. Here for the first time in HF rat models we validated the relationship between QSYQ as a whole and RAAS, and further revealed QSYQ’s specific regulation pattern by different subtypes of ATs and ACEs in RAAS.

As for the target prediction method, by our comprehensive evaluation the method integrating multiple data resources has good prediction performance, even for the target prediction of compounds only with structural information, and meanwhile its candidate target space is at the whole genome-wide scale. However, there are still some limitations in this method, for example, it assumes that each protein can bind small molecules, however actually many other factors such as druggability, cellular compartmentalization etc. also effect the binding^10^. In future, these factors will be considered to further improve the prediction efficacy.

Finally it should be noted that there are still many uncertainties in currently available data that BATMAN-TCM depends on, such as the incomplete definition of active composition of each formula, the absence of the quantitative information of components in each formula etc.. Therefore, we advise and encourage users to take full advantage of the function of user-customized input of the ingredient list, and meanwhile combine the analysis results given by BATMAN-TCM with the biological knowledge and professional experience to make the most proper judgment. Meanwhile, in future we will also regularly update formula-herb-ingredient association, known drug/ingredient-target interaction, protein interaction network, KEGG pathway data etc. BATMAN-TCM depends on, as the corresponding databases update. As the related data are continuously updated and perfected, BATMAN-TCM will perform better.

## Materials and Methods

### Target prediction

Here we used a similarity-based method to predict potential targets of TCM ingredients, the core idea of which is to rank potential drug-target interactions based on their similarity to the known drug-target interactions^22^.

Here drug-target prediction was treated as a binary classification problem, and the prediction model was constructed to distinguish true and false drug-target interaction. For each query drug-protein interaction, we defined the feature value of a classification feature as the largest one among the similarity scores between this query interaction and all known drug-target interactions (i.e. the golden standard positive dataset). The similarity between two drug-target interactions was calculated as the product of their drug similarity score and the target similarity score. Here we used 6 scores to measure the drug-drug similarity respectively based on chemical structure (including FP2 fingerprint-based^38^ and functional group-based^39^ similarity scores), side-effect, ATC (Anatomical, Therapeutic and Chemical) classification system, drug-induced gene expression and the text mining score of chemical-chemical association, and 3 scores to measure protein-protein similarity respectively based on protein sequence, closeness in a protein interaction network and Gene Ontology (GO) functional annotation^40^, resulting in 18 (=6 × 3) features in total. By minimum redundancy maximum relevance (mRMR) feature selection^41^,^42^, ultimately the prediction model integrating 8 features was used in BATMAN-TCM, including ATC-GO, FP2-closeness, STITCH-sequence, expression-closeness, ATC-sequence, functional_group-sequence, functional_group-GO and side_effect-sequence. For a query interaction, we used the maximum of the Likelihood Ratios (LRs) of the 8 features as the ultimate prediction score. LR is referred to as the ratio of the probability of feature *f* observed in the golden standard positive (GSP) dataset to that in the golden standard negative (GSN) dataset, which can measure the confidence level of a feature. Generally LR > 1 means the feature has the prediction ability^42^. In BATMAN-TCM, for a query compound, we rank all candidate proteins according to the order of the decreasing prediction scores given by the prediction model, and consider proteins with high scores as the potential drug targets of the query compound.

Here, the GSP dataset was extracted from DrugBank database (version: 20150726)^43^, including 5481 known drug-target interactions between 1204 drugs and 1212 targets, and only those interactions whose drugs were FDA-approved and had InChI structural information^44^, and whose targets were human genes/proteins were used. The GSN set with the same size was constructed by the random combination between drugs and proteins in the GSP set (excluding the GSP set).

The two structural similarity scores and the side-effect similarity score of drugs were computed following the same method as we described previously^42^. The therapeutic similarity score based on ATC classification system was computed using the method described by Zhao *et al.*^10^. Side-effect data of drugs were downloaded from SIDER database (version: Oct. 17, 2012)^45^ and known drug-ATC code associations from DrugBank (downloaded on 20150726) and KEGG (version: July 31, 2014)^46^. Gene expression profile similarity scores of drugs were directly provided by Cheng *et al.*^47^, and the text mining scores of chemical-chemical associations were downloaded from STITCH database (v3.1) which were computed based both on co-occurrence in literature and on natural language processing^48^. The 3 protein-protein similarity scores were calculated using Perlman *et al.*’s scheme^22^. Amino acid sequences of human proteins were downloaded from SwissProt database (version: 20151101)^49^, and GO functional annotations from NCBI FTP (version: 20151103)^50^. The human protein interaction network was constructed as we described previously^17^, including 102131 interactions between 11654 proteins.

### Performance evaluation of the target prediction method

“Leave-one-interaction-out” cross-validation was first used to evaluate the performance of the prediction model. In the leave-one-interaction-out cross-validation, each time one drug-target interaction was chosen from the golden standard dataset to be used as the test set, and the remaining interactions were used as the training set to train the prediction model. The score of the test drug-target interaction was computed based on the training set. A drug-target pair was predicted to be positive if its score exceeded a given cutoff. This process was repeated *N* times (*N* was the number of drug-target interactions in the golden standard dataset), and finally test sets were combined to draw the receiver operating characteristic (ROC) curve and compute the area under the curve (AUC). To further check the “*ab initio*” prediction ability of the prediction model (i.e. target prediction of drugs which haven’t any known targets), “leave-one-drug-out” cross-validation scheme was used, in which each time one drug together with its all (positive and negative) targets chosen from the golden standard dataset was used as the test set, and the remaining interactions as the training set.

The independent test set was also used to assess the efficacy of the target prediction method. The independent test positive set was composed of 4120 known drug-target interactions from KEGG database (version: July 31, 2014)^46^ after excluding the interactions in the golden standard dataset. Similarly, in order to test the “*ab initio*” prediction ability of the prediction model, we also constructed “the independent test set-drug” which was independent of the golden standard dataset not only on drug-target interactions but also on drugs. The corresponding independent test negative sets were constructed in the similar way to the GSN set’s construction, the interactions in which excluded not only those in the corresponding independent test positive set but also those in the GSP and GSN sets.

### Target analyses

To facilitate the usage, BATMAN-TCM supports users to simply input the formula/herb’s name to analyze its molecular mechanism. This function is based on formula-herb-ingredient association data from TCMID database, which covers 46914 formulas, 8159 herbs and 25210 ingredients^6^. Known targets of drugs/ingredients were from DrugBank (downloaded on July 26, 2015), KEGG (version: July 31, 2014) and TTD (Therapeutic Target Database) (version: 4.3.02)^51^. Gene Ontology (GO) functional annotations of human proteins were from NCBI FTP (version: 20111103), human biological pathway data from KEGG database (version: 20140704) and human gene-disease associations parsed from OMIM (Online Mendelian Inheritance in Man) (downloaded on Mar. 13, 2014)^52^ and TTD (version: 4.3.02). The enrichment analyses of GO functional terms, KEGG biological pathways and OMIM/TTD diseases of a group of protein targets were all based on the hypergeometric cumulative distribution test, and the multiple testing correction was based on Benjamini-Hochberg correction method^53^.

### BATMAN-TCM implementation

In BATMAN-TCM, the analysis applications of target prediction and target analyses were written in Perl, and the web presentation of the analysis results implemented in JavaScript, PHP and Cascading Style Sheet (CSS). The function of targets’ pathway mapping was implemented based on Pathview^54^, which is an R package for pathway-based data integration and visualization. The function of the visualization of ingredient-target-pathway/disease association network was implemented based on Cytoscape.js, which is a fully featured graph library written in JavaScript for network analysis and visualization^55^.

### Experimental validation

Heart failure (HF) model of Sprague-Dwaley (SD) rats was induced by direct left anterior descending coronary artery (LAD) ligation as we previously detailedly described^34^. Briefly, SD rats were anaesthetized and a left thoracotomy was performed. Then the LAD was ligated at a distance of 0.5cm from its origin. The ligated rats were randomly divided into 2 groups: 10 in the model group and 10 in the herbal group. Meanwhile, another 10 rats undergoing the same surgery but without LAD ligation constituted the sham-operated group. The herbal group was treated for 28 days using Qishen Yiqi dripping Pill (QSYQ) with a total daily dose of 0.175g/kg dissolved in water, and the model group and sham-operated group received the same volume of water. Echocardiography was used to evaluate the HF model, by which 3 parameters reflecting cardiac function were measured, including ejection fraction (EF), left ventricular end-systolic diameter (LVEDs) and left ventricular end-diastolic diameter (LVEDd).

Expression levels of Ang II (Ang II antibody, Phoenix Pharmaceuticals Inc., Germany) was detected by immunohistochemistry (IHC), ACE and ACE2 by PCR, and AT1 (ab18801, Abcam, USA) and AT2 (ab19134, Abcam, USA) by western blot.

The study was performed in accordance with the Guide for the Care and Use of Laboratory Animals published by the National Institutes of Health (NIH Publications No. 85-23, revised 1996) and was approved by the Animal Care Committee of Beijing University of Chinese Medicine.

## Additional Information

**How to cite this article**: Liu, Z. *et al.* BATMAN-TCM: a Bioinformatics Analysis Tool for Molecular mechANism of Traditional Chinese Medicine. *Sci. Rep.* **6**, 21146; doi: 10.1038/srep21146 (2016).

## Supplementary Material

Supplementary Information
